# Supplementary material for: Dynamic data-driven meta-analysis for prioritisation of host genes implicated in COVID-19
Source: Sci Rep. 2020 Dec 18;10:22303. doi: 10.1038/s41598-020-79033-3 (PMC7749145; doi:10.1038/s41598-020-79033-3)
Supplement: Supplementary file 1 — Supplementary Information 1. [file 41598_2020_79033_MOESM1_ESM.pdf]

Dynamic data-driven meta-analysis for prioritisation of host genes implicated in COVID-19

Supplementary Information

Nicholas Parkinson*,[1] Natasha Rodgers*,[1] Max Head Fourman*,[1] Bo Wang,[1] Marie Zechner,[1] Maaike C. Swets,[1] Jonathan E. Millar,[1] Andy Law,[1] Clark D. Russell†,[2] J. Kenneth Baillie†,[1] Sara Clohisey†[1]

*equal contribution

† equal contribution

Affiliations

1. Roslin Institute, University of Edinburgh, Easter Bush, Edinburgh, EH25 9RG, UK.
2. University of Edinburgh Centre for Inflammation Research, The Queen's Medical Research Institute, Edinburgh, UK.

Correspondence to j.k.baillie@ed.ac.uk and sara.clohisey@roslin.ed.ac.uk

Dynamic data-driven meta-analysis for prioritisation of host genes implicated in COVID-19

Supplementary Information Contents:

- Supplementary Table 1
- Supplementary Table 4
- Supplementary Figure S1
- Supplementary Figure S2

Supplementary Table 1: Studies and gene lists included in the meta-analysis.

Authors	Title	Year	Category	Citation
Wei *et al.*	Genome-wide CRISPR screen reveals host genes that regulate SARS-CoV-2 infection	2020	CRISPR Screen	1
Chen *et al.*	Nasopharyngeal Shedding of Severe Acute Respiratory Syndrome—Associated Coronavirus Is Associated with Genetic Polymorphisms	2006	Human Genetics	2
Ellinghaus *et al.*	Genomewide Association Study of Severe Covid-19 with Respiratory Failure	2020	Human Genetics	3
Gralinski *et al.*	Genome Wide Identification of SARS-CoV Susceptibility Loci Using the Collaborative Cross	2015	Non-Human Genetics	4
Li *et al.*	The interaction of the SARS coronavirus non-structural protein 10 with the cellular oxido-reductase system causes an extensive cytopathic effect	2005	Protein-Protein Interaction	5
Pfefferle *et al.*	The SARS-Coronavirus-Host Interactome: Identification of Cyclophilins as Target for Pan-Coronavirus Inhibitors	2011	Protein-Protein Interaction	6
Cheng *et al.*	Severe acute respiratory syndrome coronavirus protein 6 mediates ubiquitin-dependent proteosomal degradation of N-Myc (and STAT) interactor	2015	Protein-Protein Interaction	7
Huang *et al.*	Phage display technique identifies the interaction of severe acute respiratory syndrome coronavirus open reading frame 6 protein with nuclear pore complex interacting protein NPIPB3 in modulating Type I interferon antagonism	2015	Protein-Protein Interaction	8
Yu *et al.*	VHL negatively regulates SARS coronavirus replication by modulating nsp16 ubiquitination and stability	2015	Protein-Protein Interaction	9
Gordon *et al.*	A SARS-CoV-2 protein interaction map reveals targets for drug repurposing	2020	Protein-Protein Interaction	10
Jiang *et al.*	Quantitative Analysis of Severe Acute Respiratory Syndrome (SARS)-associated Coronavirus-infected Cells Using Proteomic Approaches	2005	Proteomics	11
Li *et al.*	Correlation between TGF−β1 expression and proteomic profiling induced by severe acute respiratory syndrome coronavirus papain−like protease	2012	Proteomics	12

Supplementary Table 1: Studies and gene lists included in the meta-analysis.

Akgun *et al.*	Altered molecular pathways observed in naso-oropharyngeal samples of SARS-CoV-2 patients	2020	Proteomics	[13]
Hou *et al.*	Serum protein profiling reveals a landscape of inflammation and immune signaling in early-stage COVID-19 infection	2020	Proteomics	[14]
Messner *et al.*	Clinical classifiers of COVID-19 infection from novel ultra-high-throughput proteomics	2020	Proteomics	[15]
vanderVeerdonk *et al.*	A systems approach to inflammation identifies therapeutic targets in SARS-CoV-2 infection	2020	Proteomics	[16]
Wilde *et al.*	A Kinome-Wide Small Interfering RNA Screen Identifies Proviral and Antiviral Host Factors in Severe Acute Respiratory Syndrome Coronavirus Replication, Including Double-Stranded RNA-Activated Protein Kinase and Early Secretory Pathway Proteins	2015	RNAi	[17]
Pfaender *et al.*	LY6E impairs coronavirus fusion and confers immune control of viral disease	2020	Selected Gene Screen	[18]
Li *et al.*	Virus-host interactome and proteomic survey of PMBCs from COVID-19 patients reveal potential virulence factors influencing SARS-CoV-2 pathogenesis	2020	Protein-Protein Interaction	[19]
Leong *et al.*	Microarray and real-time RT-PCR analyses of differential human gene expression patterns induced by severe acute respiratory syndrome (SARS) coronavirus infection of Vero cells	2005	Transcriptomics	[20]
Reghunathan *et al.*	Expression profile of immune response genes in patients with Severe Acute Respiratory Syndrome	2005	Transcriptomics	[21]
Baas *et al.*	Genomic Analysis Reveals Age-Dependent Innate Immune Responses to Severe Acute Respiratory Syndrome Coronavirus	2008	Transcriptomics	[22]
Yoshikawa *et al.*	Dynamic Innate Immune Responses of Human Bronchial Epithelial Cells to Severe Acute Respiratory Syndrome-Associated Coronavirus Infection	2010	Transcriptomics	[23]
Poppe *et al.*	The NF-κB-dependent and -independent transcriptome and chromatin landscapes of human coronavirus 229E-infected cells	2017	Transcriptomics	[24]

Supplementary Table 1: Studies and gene lists included in the meta-analysis.

Blanco *et al.*	Imbalanced Host Response to SARS-CoV-2 Drives Development of COVID-19	2020	Transcriptomics	25
Hadjadj *et al.*	Impaired type I interferon activity and exacerbated inflammatory responses in severe Covid-19 patients	2020	Transcriptomics	26
Langelier *et al.*	Upper airway gene expression differentiates COVID-19 from other acute respiratory illnesses and reveals suppression of innate immune responses by SARS-CoV-2	2020	Transcriptomics	27
Ravindra *et al.*	Single-cell longitudinal analysis of SARS-CoV-2 infection in human airway epithelium	2020	Transcriptomics	28
Sun *et al.*	Comparative transcriptome analysis reveals the intensive early-stage responses of host cells to SARS-CoV-2 infection	2020	Transcriptomics	29
Wilk *et al.*	A single-cell atlas of the peripheral immune response in patients with severe COVID-19	2020	Transcriptomics	30
Wyler *et al.*	Bulk and single-cell gene expression profiling of SARS-CoV-2 infected human cell lines identifies molecular targets for therapeutic intervention	2020	Transcriptomics	31

Supplementary Table 4. Manually curated genes involved in ARDS and familial HLH pathogenesis.

Gene	Syndrome	Reference
PRF1	fHLH	[32]
UNC13D	fHLH	[33]
STX11	fHLH	[34]
STXBP2	fHLH	[35]
RAB27A	fHLH	[36]
LYST	fHLH	[37]
SH2D1A	fHLH	[38]
XIAP	fHLH	[39]
MAGT1	fHLH	[40]
CD27	fHLH	[41]
AP3B1	fHLH	[42]
EGLN1	ARDS	[43]
MUC5B	ARDS	[43]
AGER	ARDS	[43]
LRRC16A	ARDS	[43]
MAP3K1	ARDS	[43]
FLT1	ARDS	[43]
IL17	ARDS	[43]
DEFB1	ARDS	[43]
FER	ARDS	[43]
ANGPT2	ARDS	[43]
IL1B	ARDS	[44]
IL6	ARDS	[44]
IL13	ARDS	[44]
F3	ARDS	[44]
COX2	ARDS	[44]
AQP1	ARDS	[44]
SERPINE1	ARDS	[44]
PLAUR	ARDS	[44]
FGA	ARDS	[44]
CEBPA	ARDS	[44]
CXCR4	ARDS	[44]
IL1R2	ARDS	[44]
TFF2	ARDS	[44]
BTG1	ARDS	[44]
GADD45A	ARDS	[44]
GJA1	ARDS	[44]
ADMR	ARDS	[44]
CCL2	ARDS	[44]
NAMPT	ARDS	[44]
THBS1	ARDS	[44]

Gene	Syndrome	Reference
GGH	ARDS	[44]
DEADH	ARDS	[44]
PI3	ARDS	[44]
FTH1	ARDS	[44]
ARL3	ARDS	[44]
BTG2	ARDS	[44]
NQO2	ARDS	[44]
CDKN1A	ARDS	[44]
PNPLA2	ARDS	[44]
NPEPL1	ARDS	[44]
CREBZF	ARDS	[44]
ANGPT2	ARDS	[44]
PPFAI1	ARDS	[44]
FAAH	ARDS	[44]
POPDC3	ARDS	[44]
IL1RN	ARDS	[44]
ARSD	ARDS	[44]
KXR3	ARDS	[44]
HCAR3	ARDS	[44]
MME	ARDS	[44]
RBP7	ARDS	[44]
UTS2	ARDS	[44]
OLFM4	ARDS	[44]
CD24	ARDS	[44]
LCN2	ARDS	[44]
BPI	ARDS	[44]
CD14	ARDS	[44]
ITGB2	ARDS	[44]
CD28	ARDS	[44]
TGFBR3	ARDS	[44]
FASL	ARDS	[44]
SOCS3	ARDS	[44]
S100A12	ARDS	[44]
IL18	ARDS	[44]
CD177	ARDS	[44]
CLEC4E	ARDS	[44]
CD300LF	ARDS	[44]
SPTPB	ARDS	[44]
ACE	ARDS	[44]
DARC	ARDS	[44]
SFTPB	ARDS	[44]
CSF2	ARDS	[44]
VEGFA	ARDS	[44]
TNF	ARDS	[44]

Gene	Syndrome	Reference
LTA	ARDS	[44]
IL10	ARDS	[44]
NFKBIA	ARDS	[44]
NFKB1	ARDS	[44]
IL8	ARDS	[44]
MYLK	ARDS	[44]
MIF	ARDS	[44]
CXCL2	ARDS	[44]
LRRC16A	ARDS	[44]
IL32	ARDS	[44]
PLAU	ARDS	[44]
MBL2	ARDS	[44]
FV	ARDS	[44]
HMOX2	ARDS	[44]
TLR1	ARDS	[44]
FTL	ARDS	[44]
TEK	ARDS	[44]
NQO1	ARDS	[44]
SOD3	ARDS	[44]
ADIPOOQ	ARDS	[44]
PPI3	ARDS	[44]
EGF	ARDS	[44]
HMOX1	ARDS	[44]
THMD	ARDS	[44]
TIRAP	ARDS	[44]
IRAK3	ARDS	[44]
FAS	ARDS	[44]
DIO2	ARDS	[44]
NFE2L2	ARDS	[44]

fHLH: Familial haemophagocytic lymphohystiocytosis (HLH) ARDS: Acute Respiratory Distress Syndrome

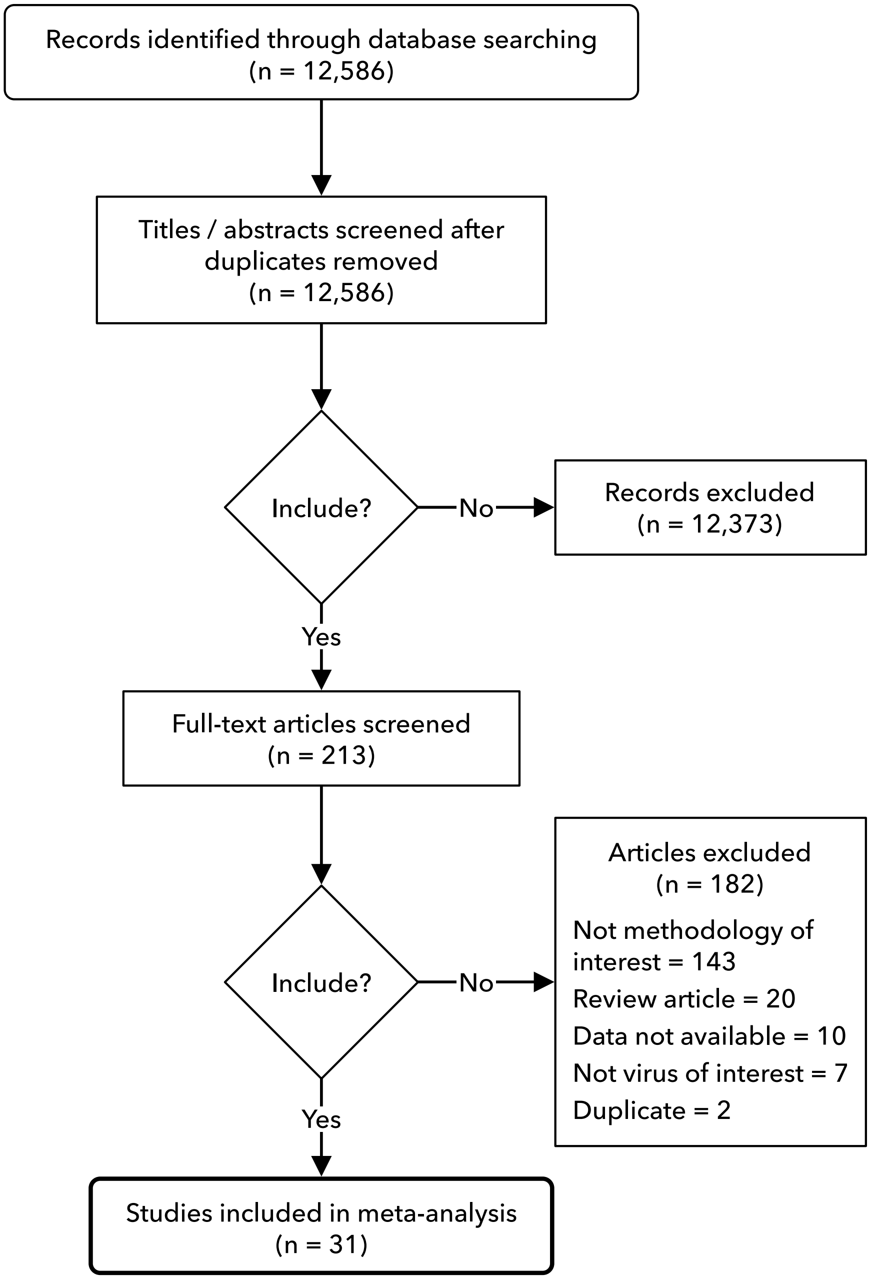

Supplementary Figure S 1: **Flow Chart of Literature Search Strategy**

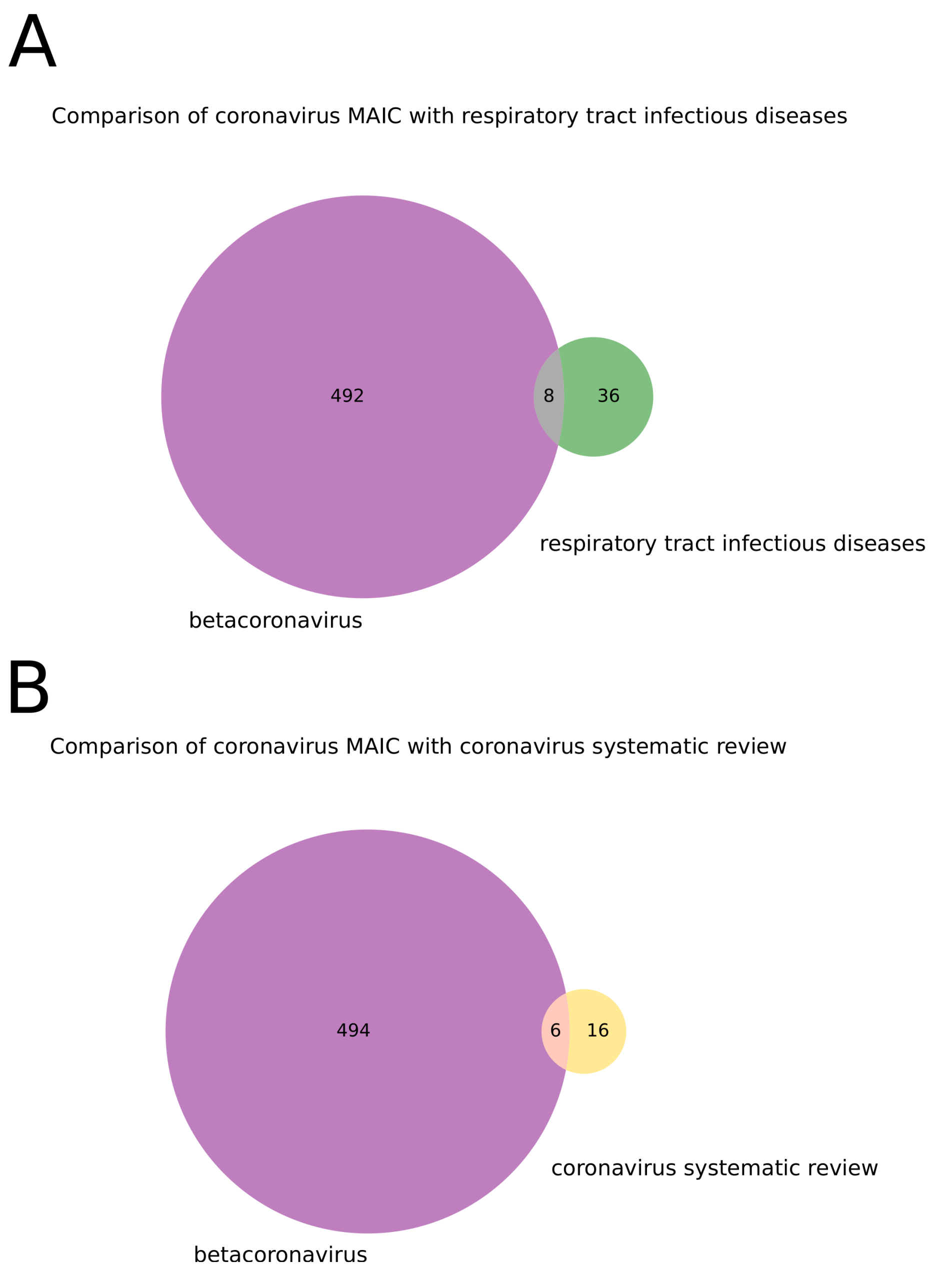

Supplementary Figure S 2: **Overlap of MAIC output with publicly available data.** A. Venn diagram of overlap between the top 500 hits from this study and a list of genes implicated in of the diseases respiratory tract caused by infections by Petarcic *et al.*[45] B.Venn diagram of overlap between the top 500 hits from this study and a list of genes assocaited with betacoronavirus infection identified in a recent systematic review by *di Maria et al.*[46]

References

1.Wei, J., Alfajaro, M.M., Hanna, R.E., DeWeirdt, P.C., Strine, M.S., Lu-Culligan, W.J., Zhang, S.-M., Graziano, V.R., Schmitz, C.O., Chen, J.S., Mankowski, M.C., Filler, R.B., Gasque, V., de Miguel, F., Chen, H., Oguntuyo, K., Abriola, L., Surovtseva, Y.V., Orchard, R.C., Lee, B., Lindenbach, B., Politi, K., van Dijk, D., Simon, M.D., Yan, Q., Doench, J.G. & Wilen, C.B. Genome-wide CRISPR screen reveals host genes that regulate SARS-CoV-2 infection. (2020).doi:10.1101/2020.06.16.155101

2.Chen, W.-J., Yang, J.-Y., Lin, J.-H., Fann, C.S.J., Osyetrov, V., King, C.-C., Chen, Y.-M.A., Chang, H.-L., Kuo, H.-W., Liao, F. & Ho, M.-S. Nasopharyngeal shedding of severe acute respiratory syndrome-associated coronavirus is associated with genetic polymorphisms. *Clinical infectious diseases : an official publication of the Infectious Diseases Society of America* **42**, 1561–9(2006).

3.Ellinghaus, D., Degenhardt, F., Bujanda, L., Buti, M., Albillos, A., Invernizzi, P., Fernández, J., Prati, D., Baselli, G., Asselta, R., Grimsrud, M.M., Milani, C., Aziz, F., Kässens, J., May, S., Wendorff, M., Wienbrandt, L., Uellendahl-Werth, F., Zheng, T., Yi, X., Pablo, R. de, Chercoles, A.G., Palom, A., Garcia-Fernandez, A.-E., Rodriguez-Frias, F., Zanella, A., Bandera, A., Protti, A., Aghemo, A., Lleo, A., Biondi, A., Caballero-Garralda, A., Gori, A., Tanck, A., Carreras Nolla, A., Latiano, A., Fracanzani, A.L., Peschuck, A., Julià, A., Pesenti, A., Voza, A., Jiménez, D., Mateos, B., Nafria Jimenez, B., Quereda, C., Paccapelo, C., Gassner, C., Angelini, C., Cea, C., Solier, A., Pestaña, D., Muñiz-Diaz, E., Sandoval, E., Paraboschi, E.M., Navas, E., García Sánchez, F., Ceriotti, F., Martinelli-Boneschi, F., Peyvandi, F., Blasi, F., Téllez, L., Blanco-Grau, A., Hemmrich-Stanisak, G., Grasselli, G., Costantino, G., Cardamone, G., Foti, G., Aneli, S., Kurihara, H., ElAbd, H., My, I., Galván-Femenia, I., Martín, J., Erdmann, J., Ferrusquía-Acosta, J., Garcia-Etxebarria, K., Izquierdo-Sanchez, L., Bettini, L.R., Sumoy, L., Terranova, L., Moreira, L., Santoro, L., Scudeller, L., Mesonero, F., Roade, L., Rühlemann, M.C., Schaefer, M., Carrabba, M., Riveiro-Barciela, M., Figuera Basso, M.E., Valsecchi, M.G., Hernandez-Tejero, M., Acosta-Herrera, M., D'Angiò, M., Baldini, M., Cazzaniga, M., Schulzky, M., Cecconi, M., Wittig, M., Ciccarelli, M., Rodríguez-Gandía, M., Bocciolone, M., Miozzo, M., Montano, N., Braun, N., Sacchi, N., Martínez, N., Özer, O., Palmieri, O., Faverio, P., Preatoni, P., Bonfanti, P., Omodei, P., Tentorio, P., Castro, P., Rodrigues, P.M., Blandino Ortiz, A., Cid, R. de, Ferrer, R., Gualtierotti, R., Nieto, R., Goerg, S., Badalamenti, S., Marsal, S., Matullo, G., Pelusi, S., Juzenas, S., Aliberti, S., Monzani, V., Moreno, V., Wesse, T., Lenz, T.L., Pumarola, T., Rimoldi, V., Bosari, S., Albrecht, W., Peter, W., Romero-Gómez, M., D'Amato, M., Duga, S., Banales, J.M., Hov, J.R., Folseraas, T., Valenti, L., Franke, A. & Karlsen, T.H. Genomewide association study of severe covid-19 with respiratory failure. *The New England journal of medicine* (2020).doi:10.1056/NEJMoa2020283

4.Gralinski, L.E., Ferris, M.T., Aylor, D.L., Whitmore, A.C., Green, R., Frie-

man, M.B., Deming, D., Menachery, V.D., Miller, D.R., Buus, R.J., Bell, T.A., Churchill, G.A., Threadgill, D.W., Katze, M.G., McMillan, L., Valdar, W., Heise, M.T., Pardo-Manuel de Villena, F. & Baric, R.S. Genome wide identification of sars-cov susceptibility loci using the collaborative cross. *PLoS genetics* **11**, e1005504(2015).

5.Li, Q., Wang, L., Dong, C., Che, Y., Jiang, L., Liu, L., Zhao, H., Liao, Y., Sheng, Y., Dong, S. & Ma, S. The interaction of the sars coronavirus non-structural protein 10 with the cellular oxido-reductase system causes an extensive cytopathic effect. *Journal of clinical virology : the official publication of the Pan American Society for Clinical Virology* **34**, 133–9(2005).

6.Pfefferle, S., Schöpf, J., Kögl, M., Friedel, C.C., Müller, M.A., Carbajo-Lozoya, J., Stellberger, T., Dall'Armi, E. von, Herzog, P., Kallies, S., Niemeyer, D., Ditt, V., Kuri, T., Züst, R., Pumpor, K., Hilgenfeld, R., Schwarz, F., Zimmer, R., Steffen, I., Weber, F., Thiel, V., Herrler, G., Thiel, H.-J., Schwegmann-Wessels, C., Pöhlmann, S., Haas, J., Drosten, C. & Brunn, A. von The sars-coronavirus-host interactome: Identification of cyclophilins as target for pan-coronavirus inhibitors. *PLoS pathogens* **7**, e1002331(2011).

7.Cheng, W., Chen, S., Li, R., Chen, Y., Wang, M. & Guo, D. Severe acute respiratory syndrome coronavirus protein 6 mediates ubiquitin-dependent proteosomal degradation of n-myc (and stat) interactor. *Virologica Sinica* **30**, 153–61(2015).

8.Huang, S.-H., Lee, T.-Y., Lin, Y.-J., Wan, L., Lai, C.-H. & Lin, C.-W. Phage display technique identifies the interaction of severe acute respiratory syndrome coronavirus open reading frame 6 protein with nuclear pore complex interacting protein npipb3 in modulating type i interferon antagonism. *Journal of microbiology, immunology, and infection = Wei mian yu gan ran za zhi* **50**, 277–285(2017).

9.Yu, X., Chen, S., Hou, P., Wang, M., Chen, Y. & Guo, D. VHL negatively regulates sars coronavirus replication by modulating nsp16 ubiquitination and stability. *Biochemical and biophysical research communications* **459**, 270–276(2015).

10.Gordon, D.E., Jang, G.M., Bouhaddou, M., Xu, J., Obernier, K., White, K.M., O'Meara, M.J., Rezelj, V.V., Guo, J.Z., Swaney, D.L., Tummino, T.A., Hüttenhain, R., Kaake, R.M., Richards, A.L., Tutuncuoglu, B., Foussard, H., Batra, J., Haas, K., Modak, M., Kim, M., Haas, P., Polacco, B.J., Braberg, H., Fabius, J.M., Eckhardt, M., Soucheray, M., Bennett, M.J., Cakir, M., McGregor, M.J., Li, Q., Meyer, B., Roesch, F., Vallet, T., Mac Kain, A., Miorin, L., Moreno, E., Naing, Z.Z.C., Zhou, Y., Peng, S., Shi, Y., Zhang, Z., Shen, W., Kirby, I.T., Melnyk, J.E., Chorba, J.S., Lou, K., Dai, S.A., Barrio-Hernandez, I., Memon, D., Hernandez-Armenta, C., Lyu, J., Mathy, C.J.P., Perica, T., Pilla, K.B., Ganesan, S.J., Saltzberg, D.J., Rakesh, R., Liu, X., Rosenthal, S.B., Calviello, L., Venkataramanan, S., Liboy-Lugo, J., Lin, Y., Huang, X.-P., Liu, Y., Wankowicz, S.A., Bohn, M., Safari, M., Ugur, F.S., Koh, C., Savar, N.S.,

Tran, Q.D., Shengjuler, D., Fletcher, S.J., O'Neal, M.C., Cai, Y., Chang, J.C.J., Broadhurst, D.J., Klippsten, S., Sharp, P.P., Wenzell, N.A., Kuzuoglu-Ozturk, D., Wang, H.-Y., Trenker, R., Young, J.M., Cavero, D.A., Hiatt, J., Roth, T.L., Rathore, U., Subramanian, A., Noack, J., Hubert, M., Stroud, R.M., Frankel, A.D., Rosenberg, O.S., Verba, K.A., Agard, D.A., Ott, M., Emerman, M., Jura, N., Zastrow, M. von, Verdin, E., Ashworth, A., Schwartz, O., d'Enfert, C., Mukherjee, S., Jacobson, M., Malik, H.S., Fujimori, D.G., Ideker, T., Craik, C.S., Floor, S.N., Fraser, J.S., Gross, J.D., Sali, A., Roth, B.L., Ruggero, D., Taunton, J., Kortemme, T., Beltrao, P., Vignuzzi, M., García-Sastre, A., Shokat, K.M., Shoichet, B.K. & Krogan, N.J. A sars-cov-2 protein interaction map reveals targets for drug repurposing. *Nature* **583**, 459–468(2020).

11.Jiang, X.-S., Tang, L.-Y., Dai, J., Zhou, H., Li, S.-J., Xia, Q.-C., Wu, J.-R. & Zeng, R. Quantitative analysis of severe acute respiratory syndrome (sars)-associated coronavirus-infected cells using proteomic approaches: Implications for cellular responses to virus infection. *Molecular & cellular proteomics : MCP* **4**, 902–13(2005).

12.Li, S.-W., Yang, T.-C., Wan, L., Lin, Y.-J., Tsai, F.-J., Lai, C.-C. & Lin, C.-W. Correlation between tgf- expression and proteomic profiling induced by severe acute respiratory syndrome coronavirus papain-like protease. *Proteomics* **12**, 3193–205(2012).

13.Akgun, E., Tuzuner, M.B., Sahin, B., Kilercik, M., Kulah, C., Cakiroglu, H.N., Serteser, M., Unsal, I. & Baykal, A.T. ALTERED MOLECULAR PATHWAYS OBSERVED IN NASO-OROPHARYNGEAL SAMPLES OF SARS-CoV-2 PATIENTS. (2020).doi:10.1101/2020.05.14.20102558

14.Hou, X., Zhang, X., Wu, X., Lu, M., Wang, D., Xu, M., Wang, H., Dai, J., Duan, H., Xu, Y., Yu, X. & Li, Y. Serum protein profiling reveals a landscape of inflammation and immune signaling in early-stage COVID-19 infection. (2020).doi:10.1101/2020.05.08.20095836

15.Messner, C.B., Demichev, V., Wendisch, D., Michalick, L., White, M., Freiwald, A., Textoris-Taube, K., Vernardis, S.I., Egger, A.-S., Kreidl, M., Ludwig, D., Kilian, C., Agostini, F., Zelezniak, A., Thibeault, C., Pfeiffer, M., Hippenstiel, S., Hocke, A., von Kalle, C., Campbell, A., Hayward, C., Porteous, D.J., Marioni, R.E., Langenberg, C., Lilley, K.S., Kuebler, W.M., Muelleder, M., Drosten, C., Witzenrath, M., Kurth, F., Sander, L.E. & Ralser, M. Clinical classifiers of COVID-19 infection from novel ultra-high-throughput proteomics. (2020).doi:10.1101/2020.04.27.20081810

16.van de Veerdonk, F.L., Janssen, N.A.F., Grondman, I., de Nooijer, A.H., Koeken, V.A.C.M., Matzaraki, V., Boahen, C.K., Kumar, V., Kox, M., Koenen, H.J.P.M., Smeets, R.L., Joosten, I., Br&uumlggemann, R.J.M., Kouijzer, I.J.E., van der Hoeven, H.G., Schouten, J.A., Frenzel, T., Reijers, M., Hoefsloot, W., Dofferhoff, A.S.M., Kerckhoffs, A.P.M., Blaauw, M.J.T., Veerman, K., Maas, C., Schoneveld, A.H., Hoefer, I.E., Derde, L.P.G., Willems, L., Toonen, E., van Deuren, M., van der Meer, J.W.M., van Crevel, R., Giamarellos-Bourboulis,

E.J., Joosten, L.A.B., van den Heuvel, M.M., Hoogerwerf, J., de Mast, Q., Pickkers, P. & Netea, M.G. A systems approach to inflammation identifies therapeutic targets in SARS-CoV-2 infection. (2020).doi:10.1101/2020.05.23.20110916

17.Wilde, A.H. de, Wannee, K.F., Scholte, F.E.M., Goeman, J.J., Ten Dijke, P., Snijder, E.J., Kikkert, M. & Hemert, M.J. van A kinome-wide small interfering rna screen identifies proviral and antiviral host factors in severe acute respiratory syndrome coronavirus replication, including double-stranded rna-activated protein kinase and early secretory pathway proteins. *Journal of virology* **89**, 8318–33(2015).

18.Pfaender, S., Mar, K.B., Michailidis, E., Kratzel, A., Hirt, D., V'kovski, P., Fan, W., Ebert, N., Stalder, H., Kleine-Weber, H., Hoffmann, M., Hoffmann, H.H., Saeed, M., Dijkman, R., Steinmann, E., Wight-Carter, M., Hanners, N.W., Pöhlmann, S., Gallagher, T., Todt, D., Zimmer, G., Rice, C.M., Schoggins, J.W. & Thiel, V. LY6E impairs coronavirus fusion and confers immune control of viral disease. *bioRxiv : the preprint server for biology* (2020).doi:10.1101/2020.03.05.979260

19.Li, J., Guo, M., Tian, X., Liu, C., Wang, X., Yang, X., Wu, P., Xiao, Z., Qu, Y., Yin, Y., Fu, J., Zhu, Z., Liu, Z., Peng, C., Zhu, T. & Liang, Q. Virus-host interactome and proteomic survey of PMBCs from COVID-19 patients reveal potential virulence factors influencing SARS-CoV-2 pathogenesis. (2020).doi:10.1101/2020.03.31.019216

20.Leong, W.F., Tan, H.C., Ooi, E.E., Koh, D.R. & Chow, V.T.K. Microarray and real-time rt-pcr analyses of differential human gene expression patterns induced by severe acute respiratory syndrome (sars) coronavirus infection of vero cells. *Microbes and infection* **7**, 248–59(2005).

21.Reghunathan, R., Jayapal, M., Hsu, L.-Y., Chng, H.-H., Tai, D., Leung, B.P. & Melendez, A.J. Expression profile of immune response genes in patients with severe acute respiratory syndrome. *BMC immunology* **6**, 2(2005).

22.Baas, T., Roberts, A., Teal, T.H., Vogel, L., Chen, J., Tumpey, T.M., Katze, M.G. & Subbarao, K. Genomic analysis reveals age-dependent innate immune responses to severe acute respiratory syndrome coronavirus. *Journal of virology* **82**, 9465–76(2008).

23.Yoshikawa, T., Hill, T.E., Yoshikawa, N., Popov, V.L., Galindo, C.L., Garner, H.R., Peters, C.J. & Tseng, C.-T.K. Dynamic innate immune responses of human bronchial epithelial cells to severe acute respiratory syndrome-associated coronavirus infection. *PloS one* **5**, e8729(2010).

24.Poppe, M., Wittig, S., Jurida, L., Bartkuhn, M., Wilhelm, J., Müller, H., Beuerlein, K., Karl, N., Bhuju, S., Ziebuhr, J., Schmitz, M.L. & Kracht, M. The nf-b-dependent and -independent transcriptome and chromatin landscapes of human coronavirus 229E-infected cells. *PLoS pathogens* **13**, e1006286(2017).

25.Blanco-Melo, D., Nilsson-Payant, B.E., Liu, W.-C., Uhl, S., Hoagland, D.,

Møller, R., Jordan, T.X., Oishi, K., Panis, M., Sachs, D., Wang, T.T., Schwartz, R.E., Lim, J.K., Albrecht, R.A. & tenOever, B.R. Imbalanced host response to sars-cov-2 drives development of covid-19. *Cell* **181**, 1036–1045.e9(2020).

26.Hadjadj, J., Yatim, N., Barnabei, L., Corneau, A., Boussier, J., Pere, H., Charbit, B., Bondet, V., Chenevier-Gobeaux, C., Breillat, P., Carlier, N., Gauzit, R., Morbieu, C., Pene, F., Marin, N., Roche, N., Szwebel, T.-A., Smith, N., Merkling, S., Treluyer, J.-M., Veyer, D., Mouthon, L., Blanc, C., Tharaux, P.-L., Rozenberg, F., Fischer, A., Duffy, D., Rieux-Laucat, F., Kerneis, S. & Terrier, B. Impaired type I interferon activity and exacerbated inflammatory responses in severe Covid-19 patients. (2020).doi:10.1101/2020.04.19.20068015

27.Mick, E., Kamm, J., Pisco, A.O., Ratnasiri, K., Babik, J.M., Calfee, C.S., Castaneda, G., DeRisi, J.L., Detweiler, A.M., Hao, S., Kangelaris, K.N., Kumar, G.R., Li, L.M., Mann, S.A., Neff, N., Prasad, P.A., Serpa, P.H., Shah, S.J., Spottiswoode, N., Tan, M., Christenson, S.A., Kistler, A. & Langelier, C. Upper airway gene expression differentiates covid-19 from other acute respiratory illnesses and reveals suppression of innate immune responses by sars-cov-2. *medRxiv : the preprint server for health sciences* (2020).doi:10.1101/2020.05.18.20105171

28.Ravindra, N.G., Alfajaro, M.M., Gasque, V., Wei, J., Filler, R.B., Huston, N.C., Wan, H., Szigeti-Buck, K., Wang, B., Montgomery, R.R., Eisenbarth, S.C., Williams, A., Pyle, A.M., Iwasaki, A., Horvath, T.L., Foxman, E.F., Dijk, D. van & Wilen, C.B. Single-cell longitudinal analysis of sars-cov-2 infection in human bronchial epithelial cells. *bioRxiv : the preprint server for biology* (2020).doi:10.1101/2020.05.06.081695

29.Sun, J., Ye, F., Wu, A., Yang, R., Pan, M., Sheng, J., Zhu, W., Mao, L., Wang, M., Huang, B., Tan, W. & Jiang, T. Comparative transcriptome analysis reveals the intensive early-stage responses of host cells to SARS-CoV-2 infection. (2020).doi:10.1101/2020.04.30.071274

30.Wilk, A.J., Rustagi, A., Zhao, N.Q., Roque, J., Martínez-Colón, G.J., McKechnie, J.L., Ivison, G.T., Ranganath, T., Vergara, R., Hollis, T., Simpson, L.J., Grant, P., Subramanian, A., Rogers, A.J. & Blish, C.A. A single-cell atlas of the peripheral immune response in patients with severe covid-19. *Nature medicine* **26**, 1070–1076(2020).

31.Emanuel, W., Kirstin, M., Vedran, F., Asija, D., Theresa, G.L., Roberto, A., Filippos, K., David, K., Salah, A., Christopher, B., Anja, R., Ivano, L., Andranik, I., Tommaso, M., Simone, D.G., Patrick, P.J., Alexander, M.M., Daniela, N., Matthias, S., Altuna, A., Nikolaus, R., Christian, D. & Markus, L. Bulk and single-cell gene expression profiling of SARS-CoV-2 infected human cell lines identifies molecular targets for therapeutic intervention. (2020).doi:10.1101/2020.05.05.079194

32.Stepp, S.E., Dufourcq-Lagelouse, R., Le Deist, F., Bhawan, S., Certain, S., Mathew, P.A., Henter, J.I., Bennett, M., Fischer, A., Saint Basile, G. de & Kumar, V. Perforin gene defects in familial hemophagocytic lymphohistiocytosis.

Science (New York, N.Y.) **286**, 1957–9(1999).

33.Feldmann, J., Callebaut, I., Raposo, G., Certain, S., Bacq, D., Dumont, C., Lambert, N., Ouachée-Chardin, M., Chedeville, G., Tamary, H., Minard-Colin, V., Vilmer, E., Blanche, S., Le Deist, F., Fischer, A. & Saint Basile, G. de Munc13-4 is essential for cytolytic granules fusion and is mutated in a form of familial hemophagocytic lymphohistiocytosis (fhl3). *Cell* **115**, 461–73(2003).

34.Stadt, U. zur, Schmidt, S., Kasper, B., Beutel, K., Diler, A.S., Henter, J.-I., Kabisch, H., Schneppenheim, R., Nürnberg, P., Janka, G. & Hennies, H.C. Linkage of familial hemophagocytic lymphohistiocytosis (fhl) type-4 to chromosome 6q24 and identification of mutations in syntaxin 11. *Human molecular genetics* **14**, 827–34(2005).

35.Côte, M., Ménager, M.M., Burgess, A., Mahlaoui, N., Picard, C., Schaffner, C., Al-Manjomi, F., Al-Harbi, M., Alangari, A., Le Deist, F., Gennery, A.R., Prince, N., Cariou, A., Nitschke, P., Blank, U., El-Ghazali, G., Ménasché, G., Latour, S., Fischer, A. & Saint Basile, G. de Munc18-2 deficiency causes familial hemophagocytic lymphohistiocytosis type 5 and impairs cytotoxic granule exocytosis in patient nk cells. *The Journal of clinical investigation* **119**, 3765–73(2009).

36.Ménasché, G., Pastural, E., Feldmann, J., Certain, S., Ersoy, F., Dupuis, S., Wulffraat, N., Bianchi, D., Fischer, A., Le Deist, F. & Saint Basile, G. de Mutations in rab27a cause griscelli syndrome associated with haemophagocytic syndrome. *Nature genetics* **25**, 173–6(2000).

37.Rubin, C.M., Burke, B.A., McKenna, R.W., McClain, K.L., White, J.G., Nesbit, M.E. & Filipovich, A.H. The accelerated phase of chediak-higashi syndrome. An expression of the virus-associated hemophagocytic syndrome? *Cancer* **56**, 524–30(1985).

38.Arico, M., Imashuku, S., Clementi, R., Hibi, S., Teramura, T., Danesino, C., Haber, D.A. & Nichols, K.E. Hemophagocytic lymphohistiocytosis due to germline mutations in sh2d1a, the x-linked lymphoproliferative disease gene. *Blood* **97**, 1131–3(2001).

39.Marsh, R.A., Madden, L., Kitchen, B.J., Mody, R., McClimon, B., Jordan, M.B., Bleesing, J.J., Zhang, K. & Filipovich, A.H. XIAP deficiency: A unique primary immunodeficiency best classified as x-linked familial hemophagocytic lymphohistiocytosis and not as x-linked lymphoproliferative disease. *Blood* **116**, 1079–82(2010).

40.Li, F.-Y., Chaigne-Delalande, B., Su, H., Uzel, G., Matthews, H. & Lenardo, M.J. XMEN disease: A new primary immunodeficiency affecting mg2+ regulation of immunity against epstein-barr virus. *Blood* **123**, 2148–52(2014).

41.Alkhairy, O.K., Perez-Becker, R., Driessen, G.J., Abolhassani, H., Montfrans, J. van, Borte, S., Choo, S., Wang, N., Tesselaar, K., Fang, M., Bienemann, K., Boztug, K., Daneva, A., Mechinaud, F., Wiesel, T., Becker, C., Dückers,

G., Siepermann, K., Zelm, M.C. van, Rezaei, N., Burg, M. van der, Aghamohammadi, A., Seidel, M.G., Niehues, T. & Hammarström, L. Novel mutations in tnfrsf7/cd27: Clinical, immunologic, and genetic characterization of human cd27 deficiency. *The Journal of allergy and clinical immunology* **136**, 703–712.e10(2015).

42.Enders, A., Zieger, B., Schwarz, K., Yoshimi, A., Speckmann, C., Knoepfle, E.-M., Kontny, U., Müller, C., Nurden, A., Rohr, J., Henschen, M., Pannicke, U., Niemeyer, C., Nurden, P. & Ehl, S. Lethal hemophagocytic lymphohistiocytosis in hermansky-pudlak syndrome type ii. *Blood* **108**, 81–7(2006).

43.Hernández-Beeftink, T., Guillen-Guio, B., Villar, J. & Flores, C. Genomics and the acute respiratory distress syndrome: Current and future directions. *International journal of molecular sciences* **20**, (2019).

44.Reilly, J.P., Christie, J.D. & Meyer, N.J. Fifty years of research in ards. Genomic contributions and opportunities. *American journal of respiratory and critical care medicine* **196**, 1113–1121(2017).

45.Patarčić, I., Gelemanović, A., Kirin, M., Kolčić, I., Theodoratou, E., Baillie, K.J., Jong, M.D. de, Rudan, I., Campbell, H. & Polašek, O. The role of host genetic factors in respiratory tract infectious diseases: Systematic review, meta-analyses and field synopsis. *Scientific reports* **5**, 16119(2015).

46.Di Maria, E., Latini, A., Borgiani, P. & Novelli, G. Genetic variants of the human host influencing the coronavirus-associated phenotypes (sars, mers and covid-19): Rapid systematic review and field synopsis. *Human genomics* **14**, 30(2020).
